# An Overview of Thermal Plasma Arc Systems for Treatment of Various Wastes in Recovery of Metals

**DOI:** 10.3390/ma15020683

**Published:** 2022-01-17

**Authors:** Sneha Samal, Ignazio Blanco

**Affiliations:** 1 FZU-Institute of Physics of Czech Academy of Science, Prague 8, Na Slovance 1999/2, 18221 Prague, Czech Republic; 2 Department of Civil Engineering and Architecture, University of Catania and UdR-Catania Consorzio INSTM, Viale Andrea Doria 6, 95125 Catania, Italy; iblanco@unict.it

**Keywords:** thermal plasma, metal, recycling, waste, minerals

## Abstract

Thermal plasma systems are being used for the recovery of metals from complex waste and minerals. The latter contain multiphase metals in various forms that are extremely tedious to separate. Thermal plasma arc melts the waste and minerals for qualitative plasma products for powder industries. In this overview, we briefly report a description of the various thermal plasma systems and their uses in recovering metal from metal-containing materials in the form of waste or minerals. Various plasma arc systems, such as transferred, nontransferred, and extended arc, have enabled the development of an efficient and environmentally friendly way to recover valuable metals from industrial wastes such as red mud and minerals such as ilmenite.

## 1. Introduction

Metal-containing materials fall into various categories, from waste materials to minerals. Metals are hidden resources in the waste materials generated from the day-to-day industrial production of human needs [1,2,3], such as metal-containing waste generated from electronic products, metal plants, minerals sectors, and metal-containing soils and minerals [4,5]. To utilize this waste in a valuable way, both hydro- and pyrometallurgical treatments have been proposed in industrial sectors. However, hydrometallurgical treatments create acidic and alkaline residual threats to the environment; as a result, the focus has been shifted more significantly toward pyrometallurgical routes. Thermal plasma is an efficient way to treat metal-containing waste materials. Plasma, generated in a highly ionized state, operates with a high energy density at high temperature. Such a system could enable the degradation of organic compounds from electronic waste materials into elemental ions and atoms, with end-products including gases [6,7,8]. Plasma waste treatment falls into various categories, such as plasma gasification and vitrification of hazardous wastes into slags. The high temperatures of the plasma melt organic and inorganic pollutants into reduced slag. The slag generated by plasma technology is very stable, less harmful, and easy to handle for further treatment. However, volatile metals do not remain in slag during plasma melting of waste material. In plasma reduction, high temperature acts as a boon for soil-containing metals in multiphase forms; for example, waste from the bauxite industry, known as red mud, and ilmenite. The different recovery methods of the valuable metals using various processes by thermal plasma are summarized in Table 1 [9,10,11,12,13,14,15]. 

Plasma is classified into two broad categories: high and low temperature. The first category comprises a completely ionized system in which the temperature reaches 10^6^–10^7^ °C and above, whilst low-temperature plasma exhibits partially ionized plasma with temperatures falling in the range of 10^1^–10^5^ °C. Low-temperature plasma is divided in two categories, thermal and nonthermal, based on the thermodynamic equilibrium temperature. Thermal plasma combines arc plasma, combustion plasma, and high-frequency plasma in its categories. Thermal plasma has wide application in treatment of wastes from wide areas, such as electronic wastes, steel-making waste, electroplating waste, etc. In the case of nonthermal plasma, generation depends on the dielectric discharge, pulsed alternating current, pulse direct current microwave, gliding arc discharge, etc. 

**Figure 1:**
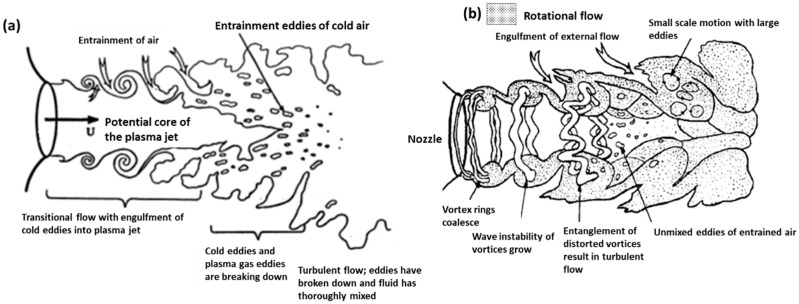
Schematic diagrams of: (**a**) transitional plasma jet; (**b**) rotational flow jet. Reprinted from [17] with permission from Elsevier 1989.

Figure 1 shows the main region in a plasma jet flow. When a plasma arc is released through the nozzle, it creates a laminar layer on the exterior layer of the arc. The inner and outer difference in velocity produces a ring vortex of flow downstream that allows the repeating of the process from the nozzle. Surroundings vortex rings collide with the main stream and form larger vortices that generate wave instability. The distorted vortex rings entangle with adjacent ones, leading to large eddies of turbulent flow. This is the entrainment of the external air, with the whole process contributing to the system’s entropy growth. Argon is considered a plasma gas, as it is able to generate of plasma. Reynolds numbers in the case of argon are lower than air in the creation of plasma [15,16].

**Figure 2:**
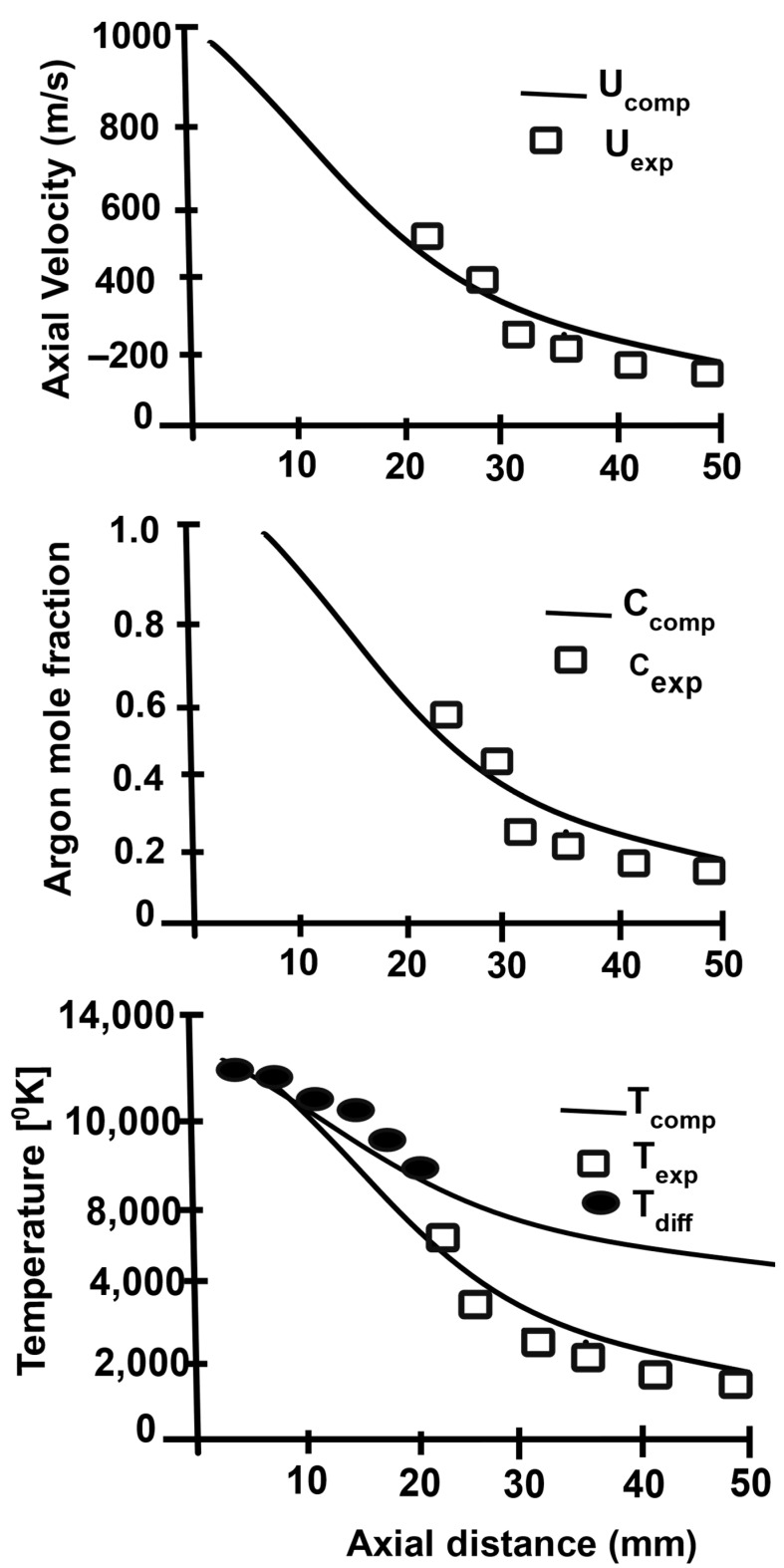
Comparison of calculated centerline fields with experimental data. Reprinted from [18] with permission from Springer 1991.

Figure 2 shows the comparison of theoretical and experimental data for the axial velocity, argon mole fraction, and plasma temperature as a function of the axial distance. A good agreement was achieved up to an axial distance of 30 mm [17,18]. 

## 2. Different Types of Thermal Plasma Arc Systems

Plasma consists of excited ions of gaseous atoms and free electrons that are able to conduct electricity. The plasma temperature can reach up to 15,000 °C or more. These temperature conditions are sufficient enough to lead to the materials’ melting and vaporization. High-temperature plasma arc can be used for several purposes, such as cutting, coating, machining, welding, heating, etc. Artificially generated plasma can be created by using a plasma-forming gas (argon, nitrogen, or hydrogen) in a gas chamber at a high flow rate (1–5 m^3^/h), and containing a tungsten electrode as the cathode of a DC power source. The positive electrode can be connected to the workpiece or to the nozzle of the gas chamber. Based on the type of connection, plasma is classified into different arc systems. Plasma is generated by ways such as thermal ionization, gas discharge, photoionization, etc. In the chemical sector, gas discharge is one of the common methods to create plasma. Plasma arc discharge is divided into various forms depending on the applied electric field and gas discharge. There are AC, DC, and RF discharge, as well as microwave discharge types. In the industry and in a semipilot plant, major three types of plasma arc are very functional, such as: (a) nontransferred arc discharge; (b) transferred arc discharge; and (c) extended arc discharge [19,20]. Waste materials in the plasma jet are carried out easily in arc discharge types. 

### 2.1. Nontransferred Arc Discharge Plasma

**Figure 3:**
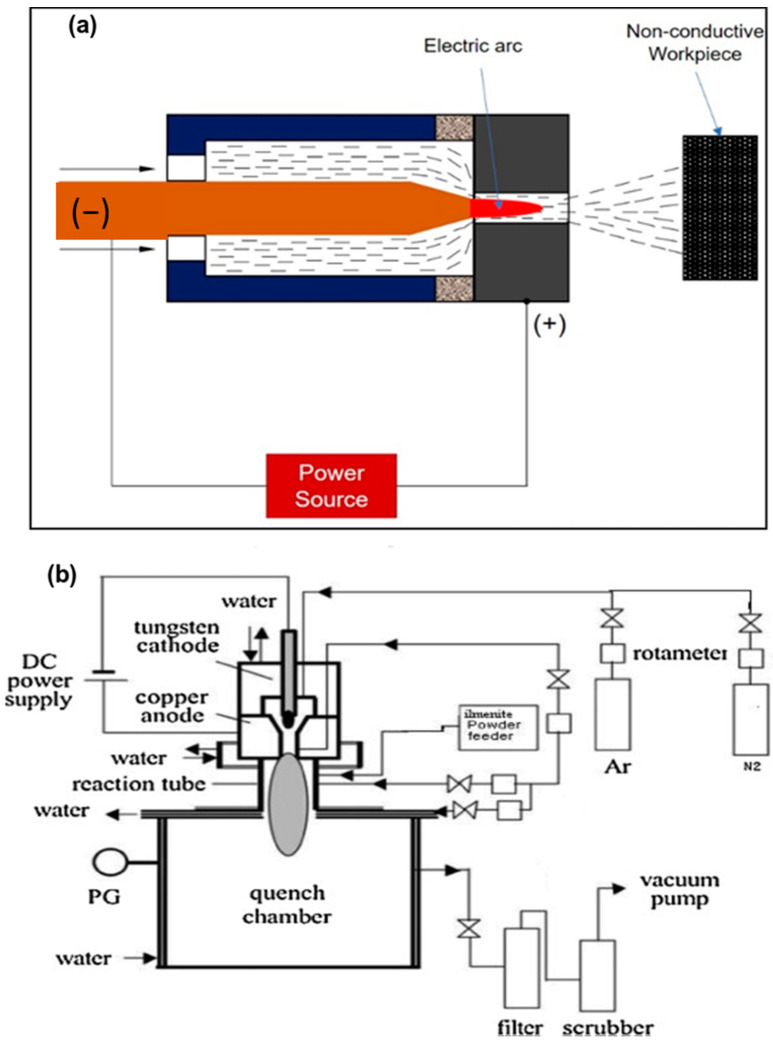
Schematic diagrams of: (**a**) nontransferred system; (**b**) nontransferred plasma reactor. Reprinted from [21] with permission from Elsevier 1989.

This typology of arc plasma refers to a nondirect plasma arc system The copper nozzle acts as a cathode with the positive terminal of the DC power source, without any connection to the workpiece. The electric arc generates between the electrode itself. The plasma gas generates the arc through the nozzle and releases with a high velocity and temperature. The maximum temperature and velocity that can be reached by a plasma jet are of 11,726 °C and 600 m/s, respectively. Different types of geometry of electrodes are used in the plasma formation in this system, with the cathode constituted by a cylindrical bar and the anode by a hollow cylinder, or at least by two coaxial cylinders. The cooling is maintained by a water-cooling system connected with electrodes. Nontransferred arc torches offer powers ranging from 1 to 6 MW. Figure 3a shows a schematization of a nontransferred arc system, whilst in Figure 3b, a reactor system is shown [21].

### 2.2. Transferred Arc Plasma System

**Figure 4:**
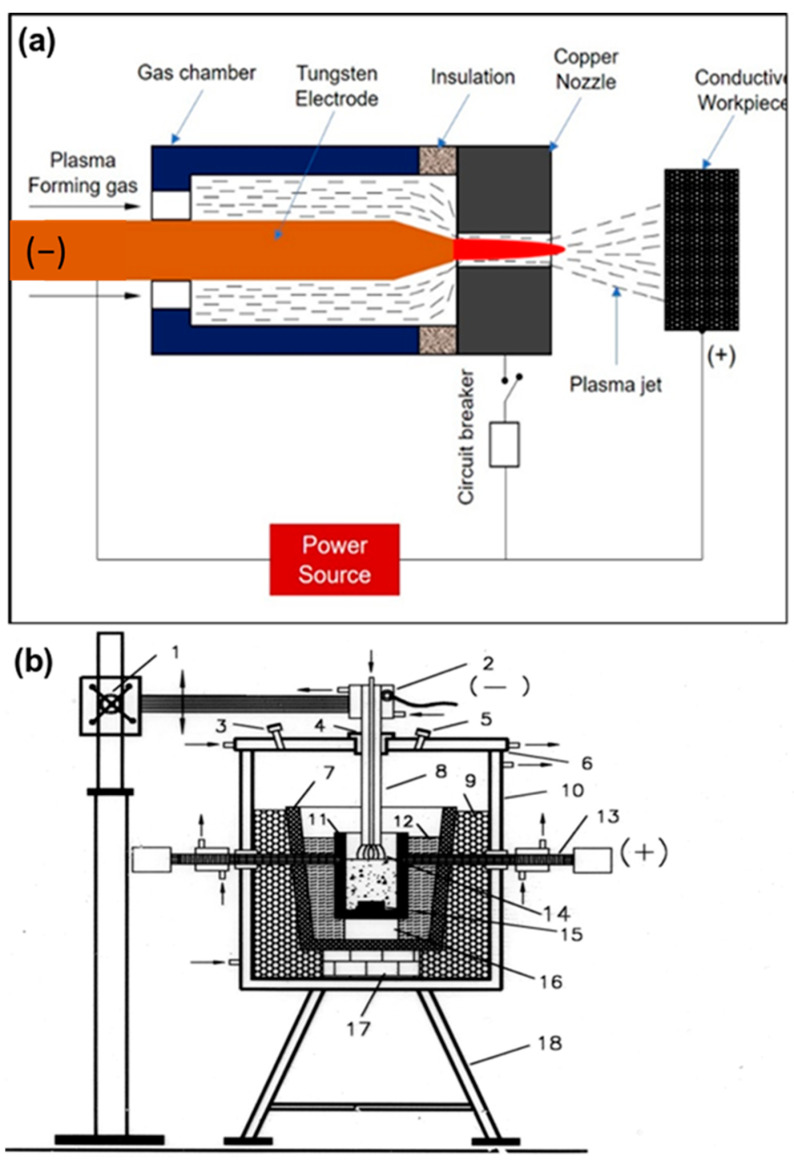
Schematic diagrams of (**a**) the transferred arc and (**b**) the corresponding plasma reactor. Reprinted from [1] with permission from Elsevier 2016. 1—Rack and pinion; 2—electrode holder with water cooling; 3—gas exhaust outlet; 4—alumina bush with graphite sleeve; 5—viewing port; 6—water-cooled steel cover; 7—Furnace hearth; 8—graphite electrode; 9—bubble alumina; 10—water-cooled steel coating; 11—graphite crucible; 12—graphite wool; 13—water-cooled graphite electrode; 14—plasma; 15—graphite block; 16—magnesia block; 17—alumina block; 18—supporting structure.

A workpiece as an integral part of the electrodes that are involved in electric circuits for the formation of plasma represents this typology, in which the workpiece and the electrode are connected, respectively, with the positive and the negative poles of a DC power source. A voltage of 200 V is applied between the cathode and anode with a maximum gap of 5–10 mm, creating an arc. The arc is more accelerated by plasma-forming gas, and is released as a plasma arc through the nozzle and strikes the workpiece. The transferred arc plasma is also called a direct plasma torch. The transferred arc reactor has heating efficiencies higher than 90%, with the limited requirement of gaseous flow for plasma generation. In Figure 4a,b, the arc and plasma reactor are schematized [22,23,24].

### 2.3. Extended Arc Plasma

**Figure 5:**
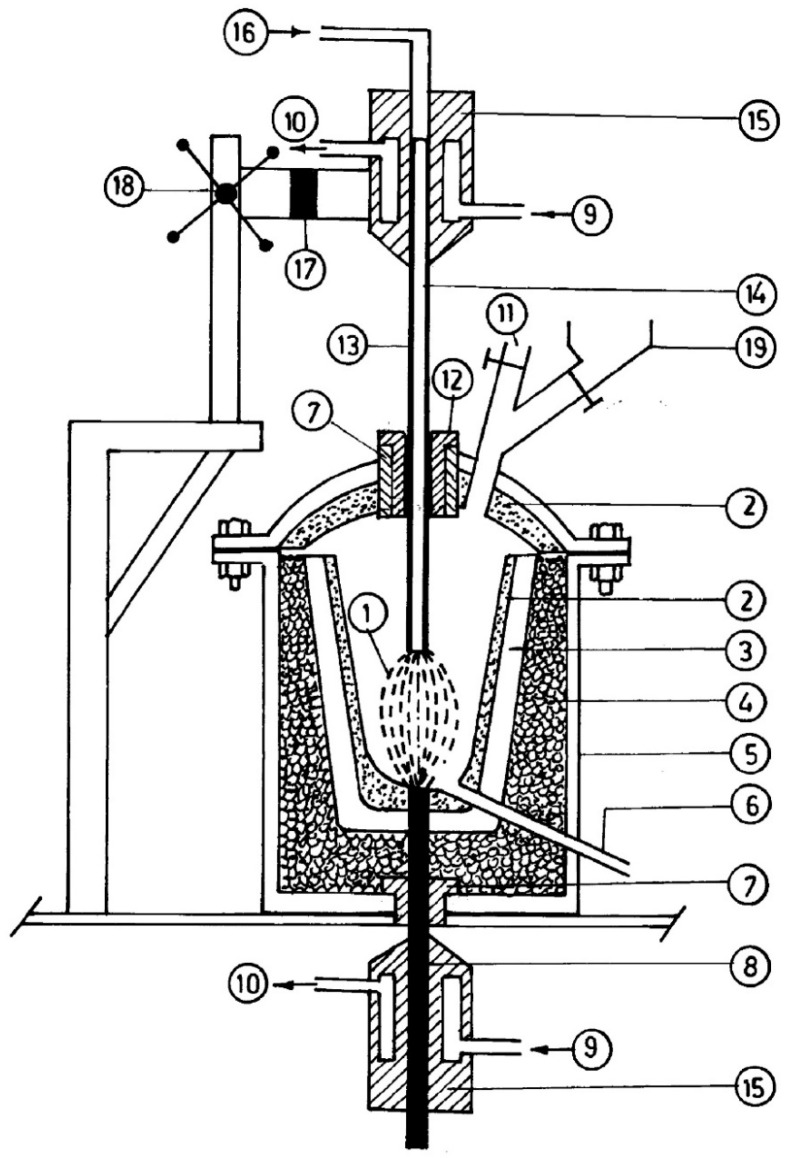
Scheme of an extended plasma reactor. Reprinted from [1] with permission from Elsevier 2016. 1—plasma arc; 2—magnesia coating; 3—graphite crucible; 4—bubble alumina; 5—M.S. casing; 6—tap hole; 7—alumina bush; 8—bottom graphite electrode; 9—water inlet; 10—water outlet; 11—outlet for exhaust gases; 12—graphite sleeve; 13—top graphite electrode; 14—axial hole; 15—copper block; 16—plasma-forming gas; 17—electrical insulation; 18—rack and pinion mechanism; 19—hopper.

Graphite electrodes, the behavior of which is more stable, allow the formation of a modified or extended arc. Usually, by using an electrode axial hole, it is possible to introduce plasma-forming argon into the arc zone, where the gas ionizes quite readily as soon as the electrodes become hot. By reducing the noise level, a considerable arc length extension is also possible. Figure 5 displays a scheme of an extended plasma reactor [25].

### 2.4. DC Plasma Reactor with Triple Cathode Arc Device

**Figure 6:**
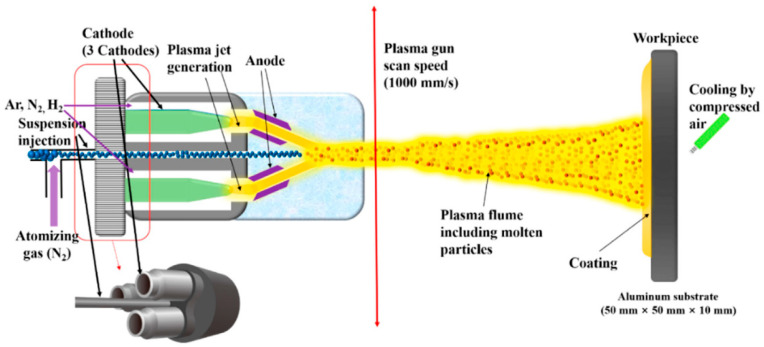
Schematic of a triple-torch plasma reactor. Reprinted from [26].

The triple-torch plasma reactor (TTPR), which is schematized in Figure 6, is extensively employed in the chemical vapor deposition method [26,27]. 

The three identical torches, operating in the range of 100 to 700 amperes, have been included in the reactor. The operating range of the arc voltage is from 28 to 53 volts when using argon or an argon/hydrogen mixture. The torches are cooled with distilled water, reaching an efficiency that falls between 45–52%, depending on the operating condition. The angle between the torch and the central line varies from 14 to 40°, with a neutral position at 30° w.r.t. the centerline. The torches can move longitudinally around the central axis by about 10 cm. A water-cooling system is supported externally to the reactor. The torch penetrates the chamber through three flexible welded metal below. The dimensions of the reactor are 50 cm in diameter and 64 cm in height, with a double walled water-cooled system. The inner chamber consists of stainless steel to avoid any corrosion. TTPR reactors are extensively used for the synthesis of fine powders such as AlN, Si_3_N_4_, and ZrC. A single torch plasma jet reactor is using liquid injection system for various areas of carbides, oxides, composites coatings. 

## 3. Plasma Treatment of Various Types of Wastes

Plasma treatment is divided into various types as follows:

(a) Plasma pyrolysis, which allows the breakdown of chemical compounds.
(b) Plasma gasification, which allows the conversion of organic compounds into a combustible gas that is generally used to generate electric power.
(c) Plasma vitrification of solid wastes, such as the hazardous waste example from alkaline red mud into neutral slag or inorganic material with hazardous metals into the ceramic matrix by adding suitable flux material, with conversion into vitrified ceramics.
(d) In combination with plasma pyrolysis and plasma vitrification or plasma gasification with plasma vitrification for organic solids.

Various categories of hazardous wastes fall under the section of plasma treatment:

- Liquids and gases containing chlorinated fluorocarbons undergo pyrolysis by the plasma process. This chemical process minimizes the formation of hazardous products. Nontransferred plasma torches are mostly used in this process.
- Solid municipal wastes, with low contamination, or hospital wastes, with high contamination, are treated by transferred arc reactors due to the high heat fluxes that allow the melting of solids.
- Incinerators residues such as fly ash, which often contain heavy metals as contaminants. These wastes are generally treated by plasma with the breakdown of metal and slag.
- Low-level radioactive wastes, asbestos, and military wastes have higher negative values and are not possible to dispose of in landfills. Plasma treatment of these wastes results in volume reduction, with the transformation of hazardous waste material into glassy slag.
- Recovery of metals from wastes such as pig iron from red mud by plasma treatment, titanium dioxide from ilmenite, platinum from discarded catalysts, and Cu foils from Cu clad plates [28,29].

## 4. Thermal Plasma Processing

Particulate materials, both solid (particles) and liquid (droplets), fed or injected into a thermal plasma arc are usually treated as the dispersed phase. A stochastic approach has been introduced using heat and mass to consider particle dispersion. A plasma jet allows the complete evaporation of wastes into ionic products, and thermal product material is obtained with subsequent quenching, which prevents undesired reaction processes [30]. 

### 4.1. Use of Thermal Plasma in Various Fields

Plasma smelting reduces the wastes and pollutants from complex compounds to simpler forms. The smelting reduction results in smaller molecules and the conversion of organic compounds to inorganic, with melted metal collected to form slag. The particles are strongly bombarded by the energetic electrons and become ionized in an excited state, thus leading to chemical reactions and the conversion from waste to simpler compounds, with residues of metal in the slag [31]. Plasma melting uses a plasma arc as a heat source for the reduction process and subsequent melting. The high temperature of the plasma flame (10,000 °C) allows metal to melt for the following separation and purification. The materials inside the plasma furnace melt and form a high-temperature molten metal pool, which is tapped in a container for cooling down into a solidified stable slag. The system consists of a plasma generator, water-cooling device, high-pressure control system, plasma-forming and carrier-gas control system, power system, and residual collection. A thermal plasma system offers feed capability; an independent energy source; high temperature, flux, and output with rapid response; low electrode consumption; and a low noise level output.

### 4.2. Thermal Plasma Technology for Waste Reduction

**Figure 7:**
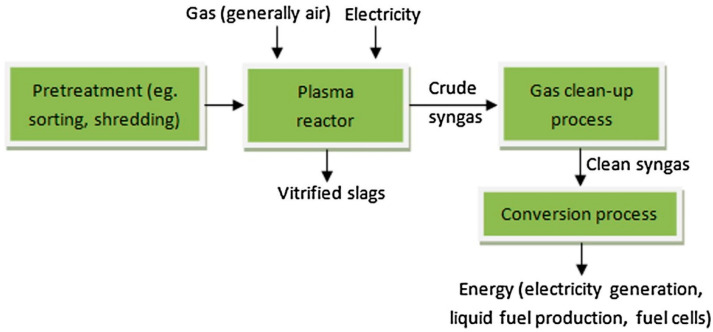
Flow chart of the plasma treatment of e-waste. Reprinted from [9] with permission from Elsevier 2016.

Waste treatment is approved via some of the consideration factors such as safety, community acceptance, and following norms of regulation. Thermal plasma has been developed as one of the efficient technologies in the reduction of various types of waste materials, such as electronics, e-waste, engineering plastic, hazardous waste, municipal solid waste, health care waste, and organic waste materials. High-temperature plasma pyrolysis allows the separation of the slag metal. The reaction kinetics speed up in the presence of plasma ions and radicals, which promote a faster reaction process and time. Plasma processing of printed circuit board waste showed metal recovery of Ag, Au, Cu, Pb, Pd, and Sn of 76%, with a more than 700 kg/day scale basis [32,33]. During the plasma melting of e-waste, a huge amount of carbon monoxide is produced, which can be considered as energy gas. Thermal plasma technology has a high economic and energy value in the sector of waste treatment. A combined process of thermal plasma technology followed by acid leaching enhanced the metal recovery step from e-waste. The extended arc plasma torch system has treated e-waste for the production of valuable metals such as Cu, Al, and Fe [34]. A schematic flow chart for the plasma treatment of solid waste is shown in Figure 7. 

**Figure 8:**
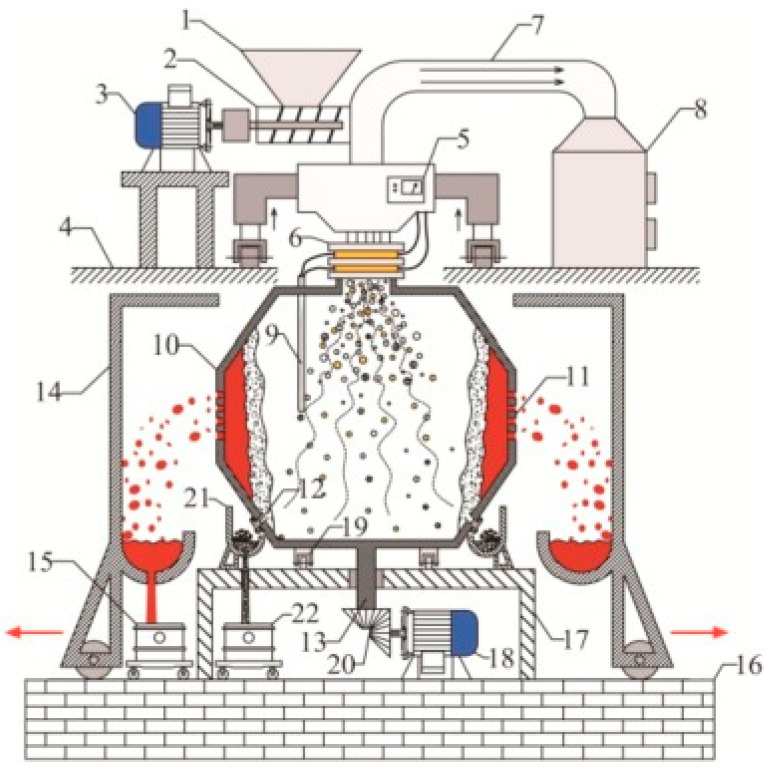
Schematic diagram of a PACT system. Reprinted from [38] with permission from Elsevier 1993. 1—Hopper; 2—spiral feeding rod; 3—motor; 4—high platform; 5—microwave generator; 6—conductive sliding ring; 7—flue pipe; 8—exhaust gas treatment tower; 9—thermocouples; 10—rotating centrifugal gravity separation reactor; 11—porous filter plate; 12—residue discharge port; 13—axis; 14—liquid metal receiver; 15—metal liquid storage tanks; 16—low foundation platform; 17—rotating centrifugal gravity separation reactor platform; 18—rotating centrifugal gravity separation reactor motor; 19—ring bearings; 20—gear; 21—slag receiver; 22—tailings storage tank.

Wastes were reduced in a plasma reactor (with melting points in the range of 1400–1600 °C), resulting in a glassy slag phase with more leaching resistance than conventional glasses. A plasma arc centrifugal treatment (PACT) system treats waste material that is fed into a sealed and water-cooled primary chamber through an air-locked system (Figure 8). A plasma torch melts the material and converts it into slag, which is collected from each subsequent interval. The rotational speed of a PACT system in the range of 15–50 rpm allows a faster speed of the materials in the system and uniform melting. Another advantage of the centrifuge system is the improved homogenization. The force exerted by the momentum of the plasma gas on the bath surface depresses the surface at the point of impingement. This process repeats 15 to 50 times per minute for the effective processing of waste material. The chemical composition of vitrified slag is uniform after molten slag cools in the mold, with small crystallite of glassy phase in the slag product. The PACT system contains a refractory liner that allows no leakage of hazardous gases to the surroundings and reduces heat loss. Since the centrifuge provides one axis of motion, the torch is usually provided with two-axis, servo-controlled positioning. Feeding chambers have isolation valves at both the inlet and outlet positions that can control any leakage from the chamber to the environment [35,36,37]. The gas/slag chamber is also air-locked for introducing molds or removing slag. The safety mold is lined with refractory material, thus ensuring a safe operation of melting inside the chamber [36,37,38]. PACT systems have been implemented for vitrifying nuclear waste in various countries, such as the USA, Switzerland, and Japan.

### 4.3. Thermal Plasma Processing of Red Mud for Recovery of Valuable Metals

Red mud is the residual chemical waste generated in the bauxite industry from alumina production. The huge quantity of waste generated during the chemical process is alkaline in nature, and disposal to the surrounding environment creates health hazards. Red mud contains various metals, with a major quantity of iron in metal and compound form [39,40]. Thermal plasma treatment for recovery of metal from red mud not only contributes to the strategy, when considering red mud as a resource, but it also will contribute less to land and health contamination. DC extended arc plasma has been implemented in the reduction of red mud toward value-added iron byproducts. Mud powder was used to recover iron. Graphite was used as a reductant with the combination of lime and low-ash coke for the reduction of red mud. In the approach of a statistical model, there is a chance of more than 75% metal recovery using a thermal plasma source [41]. The sequences of the reduction process are formulated below as a set of equations:

(1) $$3{\mathrm{Fe}}_{2}O_{3}+C\;\to 2{\mathrm{Fe}}_{3}O_{4}+\;\mathrm{CO}$$

(2) $${\mathrm{Fe}}_{2}O_{3}+3C\;\to 2\mathrm{Fe}+3\mathrm{CO}$$

(3) $${\mathrm{CO}}_{2}+C\;\to \;2\mathrm{CO}$$

(4) $$3{\mathrm{Fe}}_{2}O_{3}+\mathrm{CO}\;\to 2{\mathrm{Fe}}_{3}O_{4}+{\;\mathrm{CO}}_{2}$$

(5) $${\mathrm{Fe}}_{3}O_{4}+\mathrm{CO}\;\to 3\mathrm{FeO}+{\;\mathrm{CO}}_{2}$$

(6) $$\mathrm{FeO}+\mathrm{CO}\;\to \;\mathrm{Fe}+{\;\mathrm{CO}}_{2}$$

(7) FeO+C → Fe+ CO

(8) 14Fe3O4+CO →34Fe+ CO2

**Figure 9:**
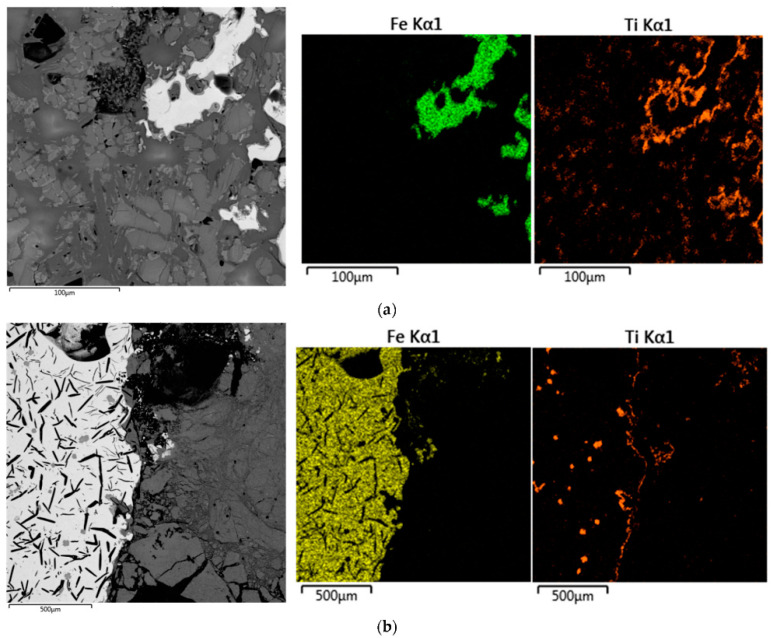

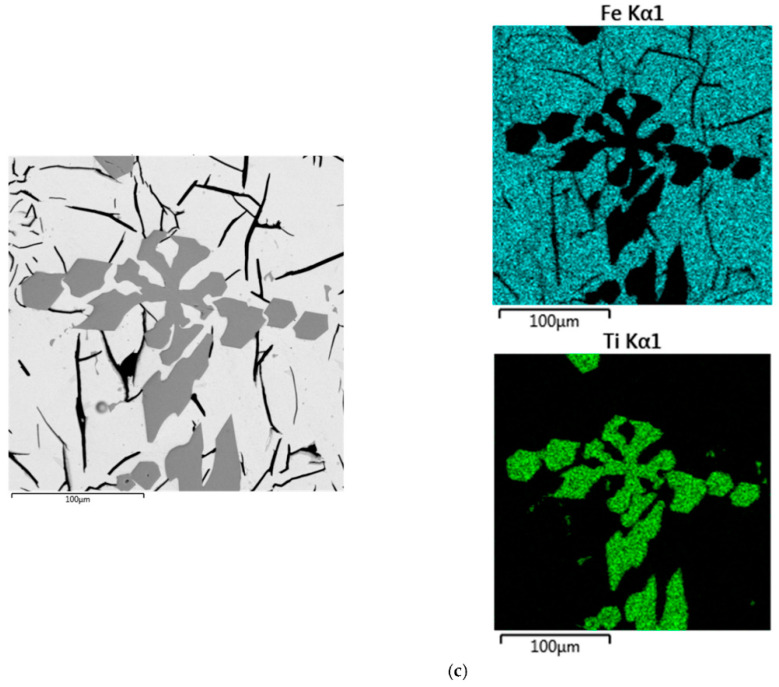
SEM images of the slag and pig iron samples: (**a**) reduction smelting at 1650 °C; (**b**) reduction smelting at 1750 °C; (**c**) titanium carbide in pig iron The mappings show the distributions of Fe and Ti. Reprinted from [35].

As red mud contains 30 to 50% iron, it undergoes a smelting reduction in a 35 kW DC extended arc thermal plasma reactor. The maximum iron recovery was 71% at an optimum smelting time of about 15 min for 350 g batch [42]. Figure 9 shows an SEM image of pig iron trapped in a reduced slag of red mud by the smelting method. The as-cast microstructures show elongated graphitic flakes dispersed in the Fe matrix (Figure 9a). In Figure 9b, an etched SEM image of an interdendritic eutectic mixture of pearlite and cementite is shown.

### 4.4. Thermal Plasma Processing of Minerals and Ores for Recovery of Metals

Thermal plasma covers a wide area in the field of mineral and ore processing in the metallurgical industry. The use of transferred and nontransferred arc plasma allows the decomposition of minerals to recover zirconia metals [43]. 

**Figure 10:**
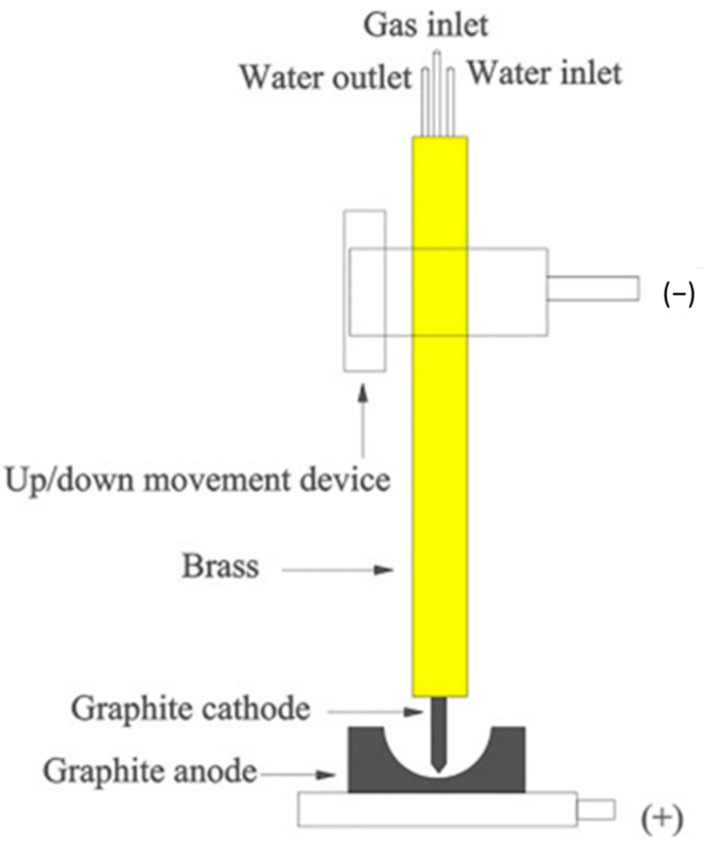
Low-pressure arc plasma. Reprinted from [43] with permission from Elsevier 1996.

**Figure 11:**
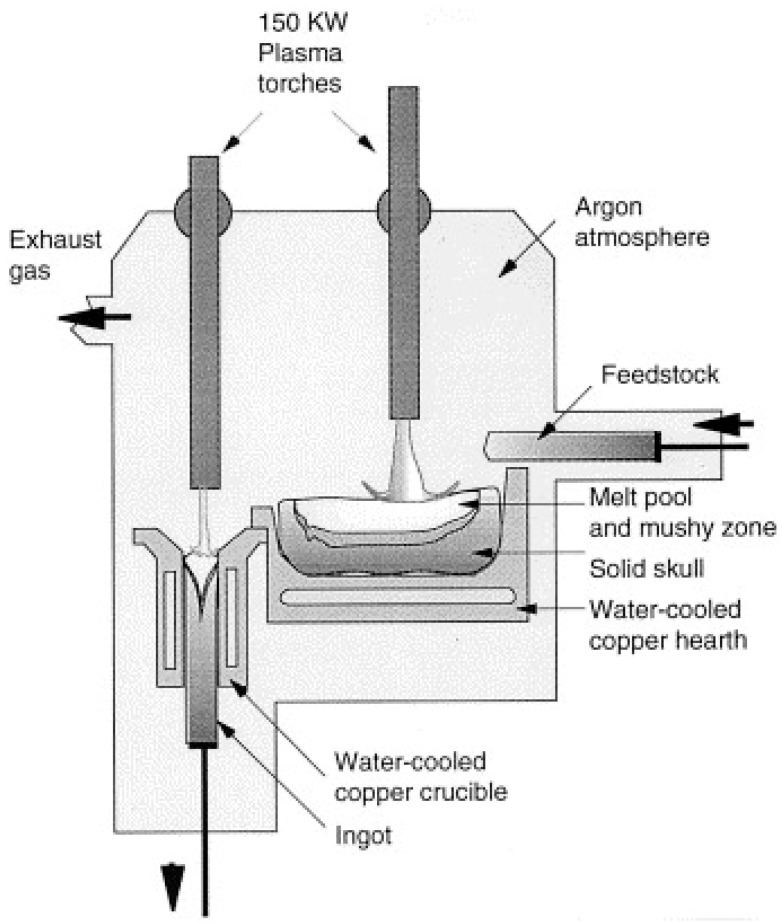
A schematic diagram of a two-torch plasma furnace withdrawing an ingot. Reprinted from [39] with permission from Elsevier 1994.

In order to proceed with zirconia recovery at low power and with a short treatment time, a low-power transferred arc plasma torch was employed. A scheme of low power transferred arc plasma is shown in Figure 10. Since the 1970s, plasma arcs have been used for recovering titanium scrap and the refining of metals, as economic value, such as the cost, is associated with base metal recovery. In the steel industry, electric arc furnaces (EAF) are widely used, and release polluted air into the surrounding areas. The plasma process has been used commercially for recovering iron and zinc from EAF dust, and for recovering aluminum from the dross generated in aluminum production, and copper alloy from brass foundry dross [44,45]. Thermal plasma is used for making titanium alloy ingots, nickel-based powders, niobium-based superconducting alloys, and several other special metal applications [36,46]. The mechanism for making ingots is represented in Figure 11 [39]. It consists of a water-cooled copper-melting hearth and water-cooled withdrawal crucibles.

Thermal plasma is also employed in material-processing industries for various phases such as metal extraction, metal refining, and alloying. Ilmenite is the major mineral resource for titanium, with the compound titanium dioxide used as white pigment in the pigment industry. Titanium dioxide pigment is used for white coloration of paints, plastics, and papers. Synthetic rutile (TiO_2_), which contains TiO_2_ in the order of 92–96%, can be produced from Ti-rich slag by various chemical routes. The more productive one is subjected to raw ilmenite, and undergoes thermal reduction treatment with the complete convertion of the iron oxide complex to a simpler form, followed by leaching with acid. The smelting process uses coal as reductant with ilmenite for reduction into titania-rich slag with 75–85% of TiO_2_ content. The technology of thermal plasma reduction has been implemented in various countries, such as Canada, South Africa, the USA, Malaysia, and India [47,48]. Transferred arc plasma technology can be used for metallurgical processing. DC thermal plasma reactors offer unique advantages for reduction processes such as high heat flux densities, with rapid start-up and shutdown times [49]. The thermal plasma process is energy-intensive; hence, slag production in countries using electric power is costly, and is not advisable. The chemical process generates a large amount of wastes that are not ecofriendly in nature, and as a result, thermal plasma has emerged as an alternative route for the production of valuable byproducts. Ilmenite (FeO.TiO_2_) is a mineral with a melting point of 1392 °C. The solid-state reaction process in thermal plasma chambers undergoes a reduction process for the formation of oxides of iron and titanium phases, as shown below:

(9) $${\mathrm{Fe}}_{2}O_{3}+C\to \;2\mathrm{FeO}+\;\mathrm{CO}\;$$

(10) FeO+C→Fe+CO

(11) $$2{\mathrm{TiO}}_{2}+C\to {\mathrm{Ti}}_{2}O_{3}+\mathrm{CO}$$

At temperatures around 1600–1700 °C, the electrothermal smelting reaction process initiates a reaction between ilmenite and reductant carbon in the formation of FeO (10–20%), Ti_2_O_3_, and TiO. A maximum value of 85–87% TiO_2_ content was produced in the slag by the thermal plasma process. The minor oxide components of Al_2_O_3_, MgO, and CaO remained the same in the slag after the reduction process.

**Figure 12:**
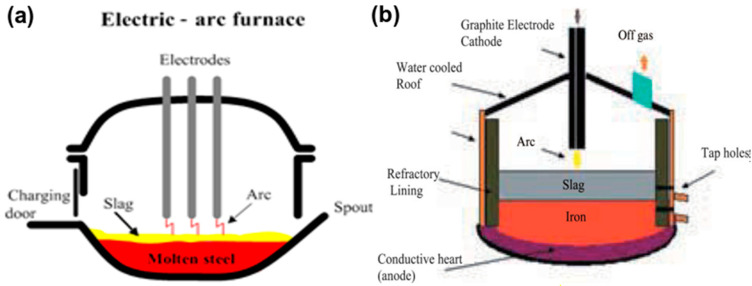
Schemes of (**a**) AC and (**b**) DC electric arc furnaces (EAFs). Reprinted from [46] with permission from Taylor & Francis 2010.

AC and DC furnaces are used for smelting of ilmenite ore. Schematic diagrams of AC and DC arc furnaces are shown in Figure 12 [46].

The parts of an AC arc furnace are the foundation, shell, chamber, bottom, and feeder. The powder is inserted by the feeder system into the chamber of the furnace. The plasma arc is established by the charging system from the rooftop. A tapping port is connected at the bottom for slag/metal collection. An electrode device, transformers with controllers for power supply and cooling, and a ventilation system are arranged in the furnace system. Three to six electrodes of the self-baking type are involved to supply electric power to the furnace system [50,51,52]. However, the two-electrode system is based on a DC furnace. The cathode and anode belong to the inside part of the system, such as the bottom of the furnace, with conducting melt forming the anode, and a hollow centrally placed electrode acting as the cathode (Figure 12b). Ilmenite and the coal mixture, along with the carrier gas, is fed into a plasma flame through the anode. This furnace can realize a more efficient energy transfer in comparison to an AC furnace. MINTEK, South Africa used a DC furnace. A water-cooled plasma arc torch was implemented in transferred mode for smelting ferrochromium. An electric arc furnace was used in Australia for smelting sand ilmenite. QIT, Canada implemented a carbothermic reduction process for making TiO_2_-rich slag [53,54]. Thermal plasma melting of ilmenite separated titania and iron into the forms of titania-rich slag and metallic iron, respectively. TiO_2_ content in the slag varied from 75–85%, whereas iron content was in the range of 10–20% in the slag. The energy consumption was found to be about 3–4 kWh for smelting 21 kg of ilmenite to slag in a static bed DC plasma reactor. However, one additional step of using metallized ilmenite reduced energy consumption significantly in the DC plasma reactor.

**Figure 13:**
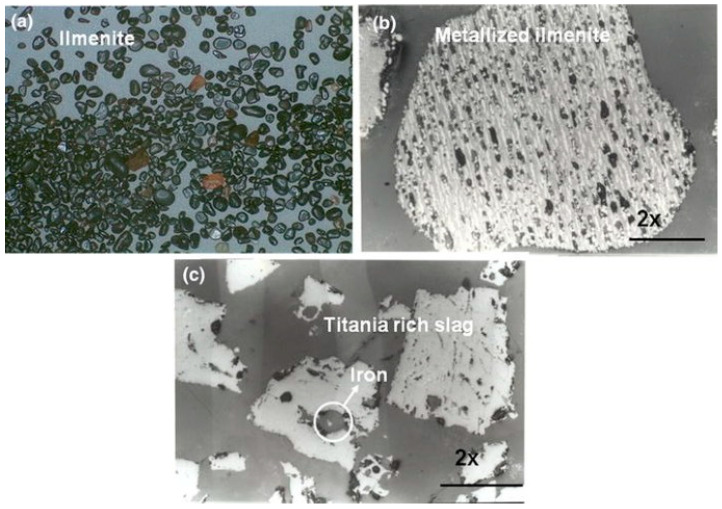
Optical microscope images of: (**a**) ilmenite; (**b**) metalized ilmenite; (**c**) Ti-rich slag with iron metal. Reprinted from [51] with permission from Springer 2016.

Figure 13 shows optical microscope images of ilmenite grains, as well as an enlarged view of the metallized ilmenite and slag produced by a thermal plasma process. Ilmenite shows grains of sand particles; in the carbothermal reduction process, it coverts into metallized ilmenite. In the metallized complex, Fe converts into a simpler form of oxide. Thermal plasma processing reduced the metallized ilmenite into Ti-rich slag, with accumulating iron as the bulk form in the slag that could be separated easily from the slag. After the separation of metallic iron, the remaining Ti-rich slag is one of the sources for the TiO_2_ pigment for industries [54]. 

**Figure 14:**
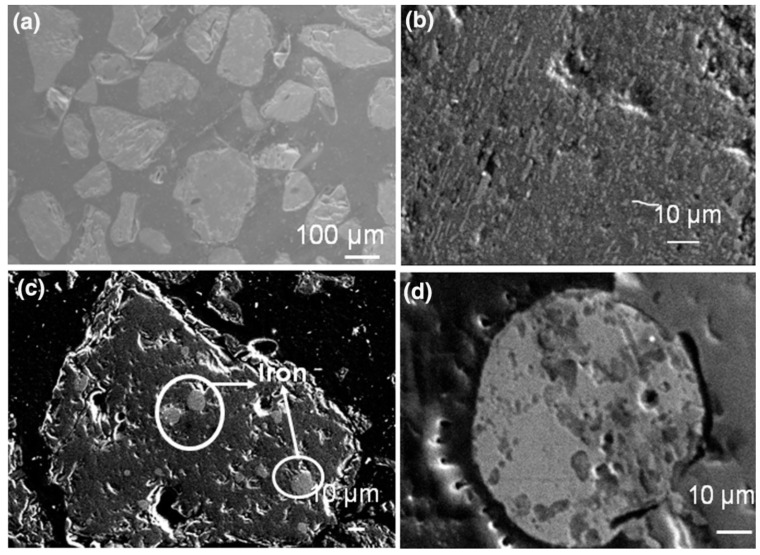
Scanning electron microscope images of: (**a**) cross-section view of ilmenite grains; (**b**) enlarged view of metallized ilmenite; (**c**) plasma product slag; (**d**) bulk iron. Reprinted from [51] with permission from Springer 2016.

Figure 14 displays a scanning electron micrograph of polished ilmenite particles with metallized ilmenite after the reduction process. Figure 14c,d show titania-rich slag, with iron entrapped with bulk iron in the more magnified image. This is one of the examples of thermal plasma processing of minerals for extraction of metals. Ilmenite dissociates into oxides of titanium and iron phases. The thermal plasma reduction process converts the iron oxide (FeO) into elemental iron (Fe) from titania-rich slag [55]. 

### 4.5. Thermal Plasma Reduction of Minerals toward Nanoparticles of Oxide

**Figure 15:**
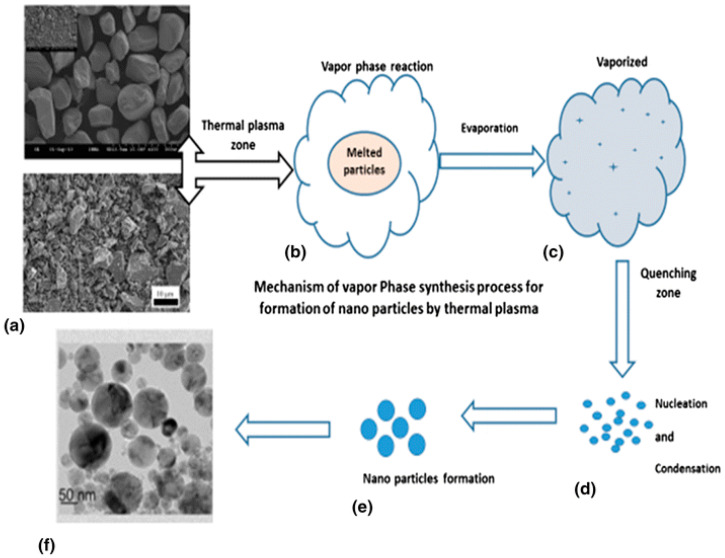
Vapor phase reduction synthesis of ilmenite particles toward titanium dioxide nanoparticles by thermal plasma process. Reprinted from [6] with permission of Springer 2018.

Recently, there has been an increasing trend toward the preparation of nanoparticles by using thermal plasma technology. Various resources such as minerals and bulk particles are considered as feedstock materials for decomposition and size reduction in nanoparticle formation. Nano-TiO_2_ particles have been generated from minerals by the vapor phase reduction method using a nontransferred arc thermal plasma reactor. This process allows dissociation of complex mineral and iron separation, with production of TiO_2_ nanoparticles as a byproduct. Figure 15 depicts a scheme of the processing method by thermal plasma, with the initial and final product. 

In addition, low-dimensional nanomaterials are one step ahead of various materials made by thermal plasma methods such as glass and alloys [15,54]. Plasma spheroidization, which allows an easy synthesis of nanoparticles attached to spherical alloy powder, is carried out by vaporization and condensation of raw powder, which undergoes a steep variation in temperature [54,55]. Spherical powder prepared by thermal plasma synthesis is not only used for powder metallurgy or additive manufacturing, but also as materials in the process synthesis of furnished coatings for surface protection [56]. 

## 5. Discussion

Thermal plasma technology has played a significant role in industrial applications, including waste material reduction, materials processing, nanopowder synthesis, deposition of functional coatings, waste treatment, metal recovery from wastes and minerals, and biomass gasification. The thermal plasma source is an integral part of the process, and is a technology for achieving various needs in the increasing demand for applications on a pilot-scale basis [57,58,59]. Waste treatment by thermal plasma technology has emerged as a value-added product. Plasma technology offers principal advantages in comparison to other existing technologies for the reduction of exhaust gas flow rates, volume reduction, lower investment costs, and faster start-up and shutdown times. Based on the above findings, it is clear that plasma gasification can decompose polypropylene granules and produce a desirable composition of syngas. Syngas could used by industries for production of heat and electricity; liquid fuel production; or for chemical products such as hydrogen, ammonia, and methanol [60,61,62]. Polypropylene granules, being the representative of plastic wastes, show the feasibility of plasma gasification to convert end-of-life plastics into commercial products with high value, and subsequently contributing to sustainable economic development [63,64]. Plasma could play a resourceful role in both the fields of waste management and energy production due to its ability to decompose waste, recover energy, and transform waste into commercial products. Plasma technology offers waste-to-energy conversion from an environmental perspective, as plasma systems could be able to decompose a wide range of waste materials and reduce hazardous emissions, due to the process occurring at high temperatures compared to the conventional methods [65]. From a social perspective, it is important to install plasma facilities in proximity to populated regions to promote better acceptance of the technology in society. In addition, some of the plasma systems enable the yielding of a vitrified slag from the inorganic components, such as ash and metals, which could be further analyzed as potential valuable byproducts. 

## 6. Conclusions

An overview of thermal plasma systems for waste treatment and metal recovery has been presented in this article. A basic understanding of thermal plasma mechanism and its application in various areas in the category of green technologies was presented here. Various types of plasma and implications toward an environmentally friendly approach to solving the problems were covered. Thermal plasma has specific advantages over combustion systems. Energy flux and the oxygen potential are not independent of temperature in a thermal plasma system. This technique, while costly due to the energy consumption of electric resources, offers a zero-waste approach to waste-disposal problems. Thermal plasma technology recovers the valuable metal resources from the minerals before discarding them as wastes to the surrounding environment. Economically, this technology contributes resources to industrial application while minimizing pollutants and wastes.

## Figures and Tables

**Table 1:** Thermal plasma methods for various metal recovery.

Methods	Materials	Thermal Plasma Methods	References
Plasma pyrolysis	Electronics waste	Treatment of waste incineration using DC nontransferred plasma	[2]
		DC transferred plasma	
		DC plasma	
Plasma arc melting and sintering	Red mud	DC extended transferred plasma reactor and furnace	[3,4,5]
	Radioactive waste	Plasma furnace	
Plasma processing and treatment	Electroplating and galvanic sludge	DC nontransferred and transferred plasma	[4,7]
Metallurgical waste decomposition to recover metals	Purification of refractory metals	Plasma arc melting furnace	[8,9,10]
	Reduction of red mud		
Plasma reduction of minerals to slag	Reduction of ilmenite	DC plasma arc furnace	[11,12,13,14]
		Plasma melting chamber	
		Nontransferred plasma	
Plasma processing of nanoparticles from minerals	Dissociation of composition and reduction of minerals	DC transferred arc plasma reactor	[15,16]

## Data Availability

Not applicable.
